# Associations between Tumor Vascularity, Vascular Endothelial Growth Factor Expression and PET/MRI Radiomic Signatures in Primary Clear-Cell–Renal-Cell-Carcinoma: Proof-of-Concept Study

**DOI:** 10.1038/srep43356

**Published:** 2017-03-03

**Authors:** Qingbo Yin, Sheng-Che Hung, Li Wang, Weili Lin, Julia R. Fielding, W. Kimryn Rathmell, Amir H. Khandani, Michael E. Woods, Matthew I. Milowsky, Samira A. Brooks, Eric. M. Wallen, Dinggang Shen

**Affiliations:** 1 College of Information Science and Technology, Dalian Maritime University, Dalian, 116023, China; 2 Department of Radiology, University of North Carolina, Chapel Hill, NC 27599, USA; 3 Department of Radiology, Taipei Veterans General Hospital, Taipei, 11217, Taiwan; 4 School of Medicine, National Yang-Ming University, Taipei, 11221, Taiwan; 5 Department of Biomedical Imaging and Radiological Sciences, School of Biomedical Science of Engineering, National Yang-Ming University, Taipei, 11221, Taiwan; 6 Department of Radiology, University of Texas Southwestern Medical Center, Dallas, TX 75390, USA; 7 Lineberger Comprehensive Cancer Center, University of North Carolina, Chapel Hill, NC 27599, USA; 8 Department of Medicine, University of North Carolina, Chapel Hill, NC 27599, USA; 9 Department of Genetics, University of North Carolina, Chapel Hill, NC 27599, USA; 10 Vanderbilt Ingram Cancer Center, Vanderbilt University, Nashville, TN 37232, USA; 11 Department of Urology, University of North Carolina, Chapel Hill, NC 27599, USA; 12 Department of Brain and Cognitive Engineering, Korea University, Seoul 02841, Republic of Korea

## Abstract

Studies have shown that tumor angiogenesis is an essential process for tumor growth, proliferation and metastasis. Also, tumor angiogenesis is an important prognostic factor of clear cell renal cell carcinoma (ccRCC), as well as a factor in guiding treatment with antiangiogenic agents. Here, we attempted to find the associations between tumor angiogenesis and radiomic imaging features from PET/MRI. Specifically, sparse canonical correlation analysis was conducted on 3 feature datasets (i.e., radiomic imaging features, tumor microvascular density (MVD), and vascular endothelial growth factor (VEGF) expression) from 9 patients with primary ccRCC. In order to overcome the potential bias of intratumoral heterogeneity of angiogenesis, this study investigated the relationship between regional expressions of angiogenesis and VEGF, and localized radiomic features from different parts within the tumors. Our study highlighted the significant strong correlations between radiomic features and MVD, and also demonstrated that the spatiotemporal features extracted from DCE-MRI provided stronger radiomic correlation to MVD than the textural features extracted from Dixon sequences and FDG PET. Furthermore, PET/MRI, which takes advantage of the combined functional and structural information, had higher radiomics correlation to MVD than solely utilizing PET or MRI alone.

As much of the focus of personalized oncology has been established on cancer characterization based on genomic and proteomic information, tumor definition is derived from invasive biopsy-based molecular assays, typically based on a single biopsy assessment. However, assessing the landscape of molecular features has been challenged by the issue of intratumoral heterogeneity, as highlighted by the pivotal examination of Gerlinger, *et al*.^1^, which demonstrated striking genetic heterogeneity in clear cell renal cell carcinoma (ccRCC). Addressing this tumor heterogeneity for appropriate patient assessment and assignment to appropriate therapy has become a crucial issue in the field of oncology. As demonstrated and confirmed in the subsequent studies^2^,^3^, ccRCCs have extraordinarily spatial and temporal heterogeneity that makes single biopsy limited to reflect the entire tumor genomic picture. Radiomics provides a new approach of characterizing and quantifying medical imaging data and adds a new dimension for personalization of cancer management^4^,^5^,^6^. Radiomics builds a high-dimensional mineable feature space and also quantitative tumor phenotypes by using automated high-throughput extraction of quantitative features of medical images^7^. And several radiomics-based predictive models have been built for various clinical factors (i.e., tumor grades, survival outcomes, treatment response, etc.)^7^. The opportunity to assess heterogeneous features, beyond the limitations of a single biopsy, and also to re-evaluate these features over time, is an essential next step in precision medicine disease evaluations.

Tumor angiogenesis is an essential process for tumor growth, proliferation and metastasis, and is also an important prognostic factor of ccRCC and plays a role in guiding treatment^8^,^9^. The vascular endothelial growth factor (VEGF) and its receptors have been established as key promoters of vascular bed expansion^10^ and are aberrantly expressed in ccRCC. VEGF and VEGFR receptors have been targeted for anti-angiogenesis therapy, with several VEGF-targeted agents approved by the FDA to treat metastatic renal cell carcinoma^11^ These therapies can cause both morphologic changes, such as necrosis or hemorrhage, and functional changes, such as reduced tumor vascularity, and also cause vasculopathy of medium/large vessels in the treated ccRCCs^12^,^13^. However, the current method of standard response criteria is based on measurement of tumor diameters, and is limited in monitoring the effect of targeted therapy^14^ Tumor angiogenic activity may be assessed with direct and indirect methods, including the commonly used measurement of tumor microvascular density (MVD), which is a density calculation of vessels in tissue based on immunohistochemical staining of endothelial cells. VEGF is a key mitogen that promotes the expansion of vascular networks, and locally corresponds closely with levels of MVD^15^. Compared with the immunohistochemical stains and microarray analysis, which require invasive tissue sampling and are difficult to repeat, medical imaging is noninvasive and routinely used in clinical practice. This noninvasive method to measure the tumor vascularity could be valuable to monitor the treatment response of targeted therapy agents. Several imaging studies have attempted to find the associations between imaging features and tumor angiogenesis, but most of them only focused on temporal-related techniques, such as dynamic contrast enhancement or blood flow imaging, obtained from one single imaging modality^16^,^17^,^18^. To leverage the emergence of the hybrid positron emission tomography/magnetic resonance imaging (PET/MRI) systems, which offers combined anatomical details provided by MR imaging and metabolic information from PET, the overarching goal of this study is to test the feasibility of measuring microvascular density of ccRCCs by using PET/MRI radiomics. We also investigated the added values of PET/MRI by conducting a comparison among spatiotemporal features of multiphasic dynamic MRI, additional textural features of Dixon, and glucose metabolic activities of PET.

## Methods

### Patient Enrollment

This HIPPA-compliant study was reviewed and approved by the institutional review board of University of North Carolina. The methods used in this study were carried out in accordance with the Declaration of Helsinki. Written informed consent was obtained from each patient prior to study initiation. The inclusion and exclusion criteria, protocols of imaging acquisition and tumor specimens processing had been published in another previous study^19^.

Between 2012 and 2013, a total of 18 patients were recruited and imaged, of which 10 patients were histologically confirmed with ccRCC. We used 9 of these 10 patients (5 men; mean age, 55.6 ± 12.5 years) for further radiomic analysis in this study, because one patient had suboptimal imaging quality.

### PET/MRI Protocol and Region-of-Interest (ROI) Placement

**Figure 1:**
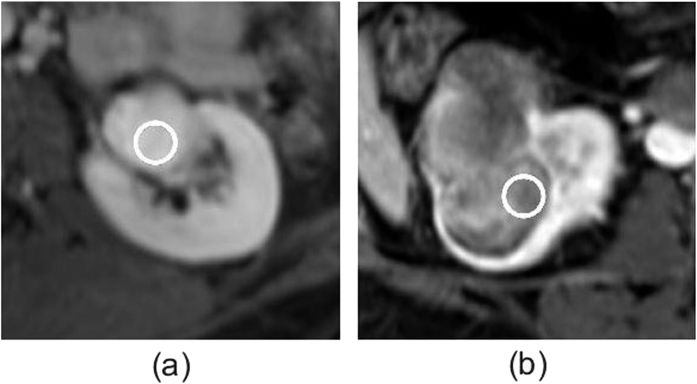
Two illustrative cases for selection of ROI location (~1 cm diameter). (**a**) A ROI was placed in the well-enhancing part in left renal clear cell carcinoma in a 37-year-old female patient. The total microvascular density was 313.08/mm^2^. (**b**) A ROI was placed in left renal cell carcinoma in a 46-year-old female patient. This part was only slightly enhanced. The total microvascular density was 54.74/mm^2^.

All patients followed a standardized PET/MRI protocol by using a hybrid PET/MRI scanner (Biograph mMR, Siemens Healthcare, Erlangen, Germany) as reported in our previous study^19^. In addition to the standard PET protocol and MR attenuation correction sequence, the MRI protocol also included three-dimensional (3D) Dixon sequence and a series of breath-hold dynamic contrast enhancement (DCE) sequence using 3D fat-saturated VIBE (volumetric interpolated breath-hold examination) at the corticomedullary, nephrographic and excretory phases with delay times of 25 s, 75 s, and 180 s, respectively (with the detailed parameters of 3D sequences provided in the Supplementary Table S1). In order to investigate the potential links between intratumoral angiogenic expressions and image features, a team of experienced radiologists, nuclear medicine specialists, and surgeons collaborated pre-operatively to place several ROIs in different parts of tumors on PET/MRI. Pre-contrast and three post-contrast sets of DCE images were then evaluated together. The post-contrast set exhibiting the signal changes was finally chosen, overlaid with PET, and visually evaluated. Several ROIs (~1 cm diameter) were defined in the regions with FDG uptake, where each defined ROI had internal uniform contrast enhancement. These defined ROIs were transferred to the operation room for tissue sampling after total nephrectomy under supervision of a board certified radiologist^19^. Figure 1 shows two illustrative cases of excretory phases of DCE images for ROI placement.

### Microvascular Density Measurement

Tumor samples were portioned for snap freezing in liquid nitrogen or formalin fixed and paraffin embedded. Definiens Tissue Studio software (Munich, Germany) was used to measure the MVD in the CD31 stained slides. The definition of MVD in this study is the count of Microvessel per unit of area rather than the area of Microvessel, as the anti-angiogenic therapy has been shown to exert different influence between small and medium/large vessels by examining the post-treated tumor specimens^12^. Therefore, the MVD was also divided into small (50–100 μm^2^), median (100–1000 μm^2^) and large-sized (greater than 1000 μm^2^) vessels according to the measured sizes. Any vessels with an area less than 50 μm^2^ were excluded because CD31 can stain more than just vessel endothelium, such as neutrophils, macrophages, platelets, etc. It is difficult to distinguish microvessel and individual cells or cell fragments that are less than 50 μm^2^.

### VEGF Analysis

mRNA was extracted from fresh frozen tissue specimens using the Qiagen AllPrep DNA/RNA Mini Kit (Valencia, CA), amplified, labeled and hybridized against a reference on Agilent Whole Human Genome (4 × 44k) Oligo Microarrays, as previously described for all specimens with suitable quality RNA^19^. The expressions of overall VEGF, and members of VEGF family, including VEGF-A, VEGF-B, VEGF-C, and VEGF receptor (VEGF-R), were used for the subsequent analyses.

### Imaging Realignment and Co-registration

We included four sets of high-resolution 3D images for radiomics analysis, including multiphasic dynamic contrast enhancement (DCE), Dixon fat images (Dixon_F), Dixon water images (Dixion_W), and 2-[fluorine-18]fluoro-2-deoxy-D-glucose (^18^F-FDG) PET. PET images were converted to standardized uptake value (SUV) maps^20^,^21^,^22^. In the following section, PET image means the SUV map.

**Figure 2:**
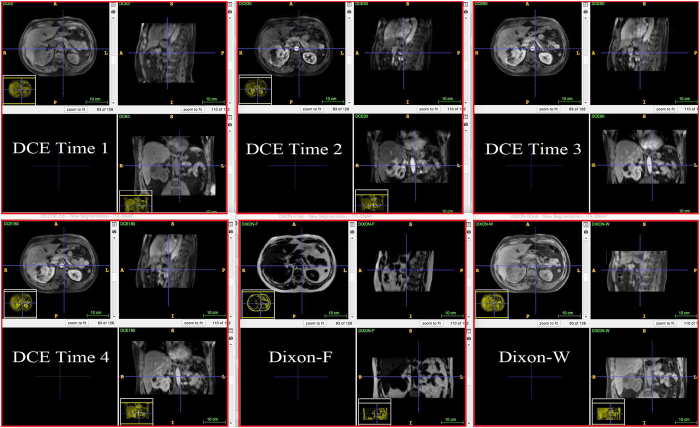
The multimodal intra-subject registered results of subject 1 except PET. There are 6 red boxes, which are divided into 2 rows and 3 columns. Each box is obtained from ITK-SNAP, and composed of 4 sub-figures: top left sub-figure is about axial plane, top right sub-figure about sagittal plane, bottom right about coronal plane. In the top row, the left box is about DCE time 1 (pre-enhanced), the middle one is about DCE time 2 (postenhanced), the right one about DCE time 3 (postenhanced); in the bottom row, the left box is about DCE time 4 (postenhanced), the middle one about Dixon_F, and the right one about Dixon_w.

Prior to texture analysis, the multimodal intra-subject registrations were conducted. For each subject, PET images and MR images, including DCE, DIXON_F and DIXON_W, were aligned to the pre-contrast scout Dixon images for structural image registration and motion correction. The FLIRT tool (FMRIB’s Linear Image Registration Tool), a fully automated robust and accurate tool, was used for linear (affine) intra- and inter-modal image registration, which has been extensively and quantitatively evaluated on many kinds of image modalities including PET^23^,^24^,^25^,^26^. In particular, in this study, theo FLIRT tool (FSL v5.0) was used to implement the 3D affine registration with 12 parameters. The cost function was the based on the maximization of mutual information, and the interpolation was trilinear. Experienced and trained radiologists visually checked the registered images in consensus, and confirmed FLIRT to be helpful for structural image registration and motion correction. The registration results for subject 1 were shown in Fig. 2, except PET due to limited display space.

### Radiomic Features Extraction

A total of 168 radiomic features were extracted automatically by an in-house tool implemented in Matlab 2014a (MathWorks Inc.) from MRI, including DCE, Dixon_F, and Dixon_W and PET images, which were defined in Supplementary Method and listed in Supplementary Table S2^27^,^28^,^29^,^30^. These radiomic features described the tumor characteristics and can be divided into two groups: Group 1, spatiotemporal association features (ST_F); Group 2, texture features (TEX_F). Group 1 included 42 features extracted from the time-series modality DCE-MRI, which represented the tumor spatiotemporal enhancement patterns. Group 2 consisted of 3 sets of 42 textural features extracted from the non-time-series images (i.e., Dixon_F, Dixon_W, and PET) respectively, in which the textural features quantified intra-tumor heterogeneity differences in the textures of tumor volume contained within each ROI.

### Analysis of Sparse Canonical Correlation

In multivariate analysis, a proper method to inspect the relationship between two sets of variables based on their correlation is canonical correlation analysis (CCA), which determines linear combinations of variables for each data set such that the two linearly combined data sets have maximum correlation^31^. But, CCA usually uses all or most of variables from each data set because its focus is on the relationships between variables in different data sets rather than the relationships within each data set, and thus hinders the clinical interpretation and biomarker discovery and validation; furthermore, it is not applicable computationally for the case of high dimensional data and small samples, since it is difficult to calculate the inverse of covariance matrix of data sets when the number of variables in data sets surpasses the number of samples, which is common in radiomic studies. Therefore, in this study, sparse canonical correlation analysis (SCCA)^32^,^33^,^34^ is performed to overcome the above disadvantage and obtain the correlated sets of variables that are sufficiently small for biological interpretation and further investigation. SCCA seeks sparsity in both sets of variables simultaneously. It incorporates variable selection and produces linear combinations of small subsets of variables from each group of measurements with maximal correlation. The corresponding patterns of non-zero coefficients can be interpreted in terms of feature expression that shows similar effects across data sets^35^.

Suppose that we have *M* observations of the paired variables 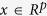 and 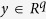. Denote 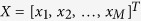 as the *M* × *p* matrix for the first set of variables; and 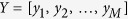 as the *M* × *q* matrix for the second set of variables. The SCCA with *l*_1_-norm regularization is used to penalize the ordinary CCA by the sum of the absolute value of weight vector. We assume that the columns of *X* and *Y* have been centered to have zero mean and normalized to have unit variance. The SCCA can then be formulated as 
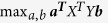
 subject to 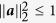, 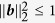, 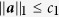, 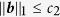.

Where parameters *c*_1_ and *c*_2_ indicate the sparseness of the projection vectors (or weight vector) ***a*** and ***b***, respectively, and adjust the amount of shrinkage. We refer to *X**a*** and *Y**b*** as the canonical variables. Since the maximum number of canonical variables is 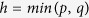, the projection matrices are defined as 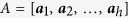, 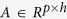, and 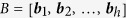, 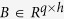, where each column contains the projection vector that is associated with a canonical variable. The pair of ***a***_*i*_ and ***b***_*i*_ is the *i*th canonical components for *X* and *Y*, corresponding to the *i*th largest correlation. The *i*th largest correlation coefficient between *X* and *Y* is computed as 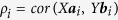. Here, 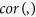 denotes the Pearson correlation function. In this study, Spearman correlation is also used, in addition to Pearson correlation, because most other leading studies using radiomics to correlate with tumor phenotype have demonstrated that imaging features tend to have a nonparametric relationship with biological parameters^36^,^37^,^38^.

To identify the latent pairwise relationships among the radiomic features, microvascular density and VEGF, we constructed three data set matrices: (1) *RF* for radiomic features; (2) *MVD* for microvascular density; (3) *VEGFset*. A row of *RF* consists of 168 dimensions, involve 42 dimensional ST_F and 126 dimensional TEX_F. MVD matrix include 4 features, i.e., total MVD, and MVD of small-sized, middle-sized, large-sized vessels respectively. *VEGFset* contains 5 variables, VEGF, VEGF-A, VEGF-B, VEGF-C and VEGR-R. So, there are 2 pairs of relationships to be analyzed. (1) *RF* and *MVD*, where we obtain four pairs of canonical weight vectors, each pair of which have a canonical correlation coefficient. (2) *RF* and *VEGFset*, where we obtain 5 pairs of canonical weight vectors and also 5 corresponding correlation coefficients.

### Identifying PET/MRIFeatures Associated with Angiogenesis

In order to compare the contributions of different sets of imaging features, we grouped the features into 4 sets to simulate possible clinical situations: DCE alone (only using features of DCE images), MRI alone (combined DCE features and Dixon features), PET alone (only using features of FDG PET), and PET/MRI (combined all features), where we performed the SCCA analysis respectively.

## Results

The mean tumor size was 8.2 ± 4.8 cm (ranging 3.5–17 cm) and the median Fuhrman grade was 2 (ranging 2–4). The median clinical AJCC staging was 2 (ranging 1–4). In total, 43 tumor samples were collected from the 9 ccRCC tumors, with a range of 3–6 subsamples taken from each tumor. MVD were successfully obtained from all specimens (159 ± 94.7/mm^2^), and expressions of VEGF family and VEGFR were obtained from 23 of the specimens.

### Radiomic Analysis

We performed pairwise relationships of sparse canonical correlation analysis between radiomics features, MVD and VEGFset.

### Sparse Canonical Correlation Analysis between RF and MVD

**Figure 3:**
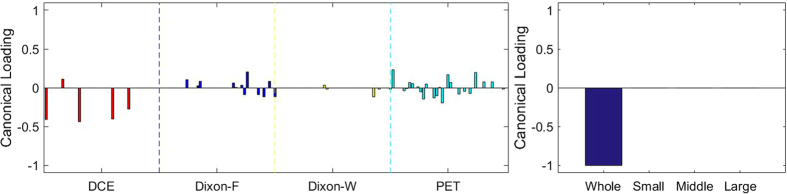
The first canonical component for sparse canonical correlation analysis between microvascular density and radiomic features (*p* = 0.044 for z-stat) with spearman correlation coefficient *ρ*_1_ = 0.466.

**Figure 4:**
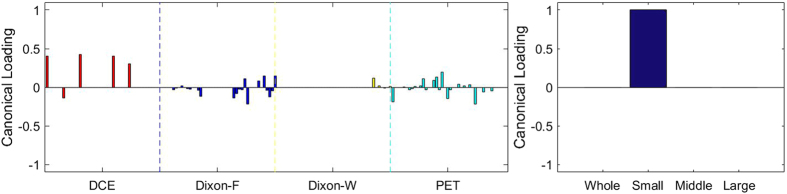
The second canonical component for sparse canonical correlation analysis between microvascular density and radiomic features (*p* = 0.044 for z-stat) with spearman correlation coefficient *ρ*_2_ = 0.375.

**Figure 5:**
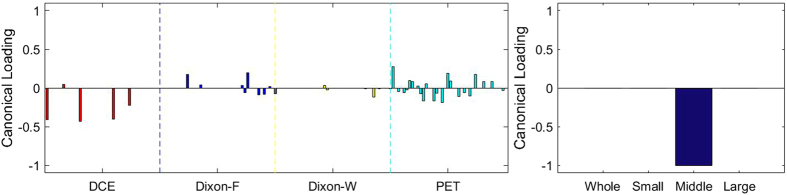
The 3^rd^ canonical component for sparse canonical correlation analysis between microvascular density and radiomic features (*p* = 0.044 for z-stat) with spearman correlation coefficient *ρ*_3_ = 0.513.

**Figure 6:**
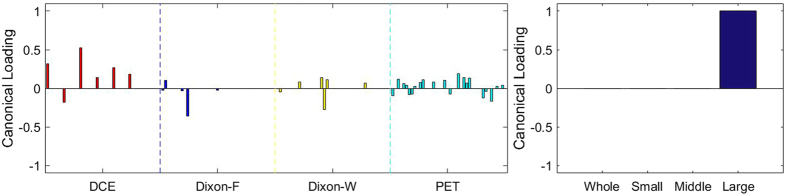
The 4^th^ canonical component for sparse canonical correlation analysis between microvascular density and radiomic features (*p* = 0.044 for z-stat) with spearman correlation coefficient *ρ*_4_ = 0.656.

**Figure 7:**
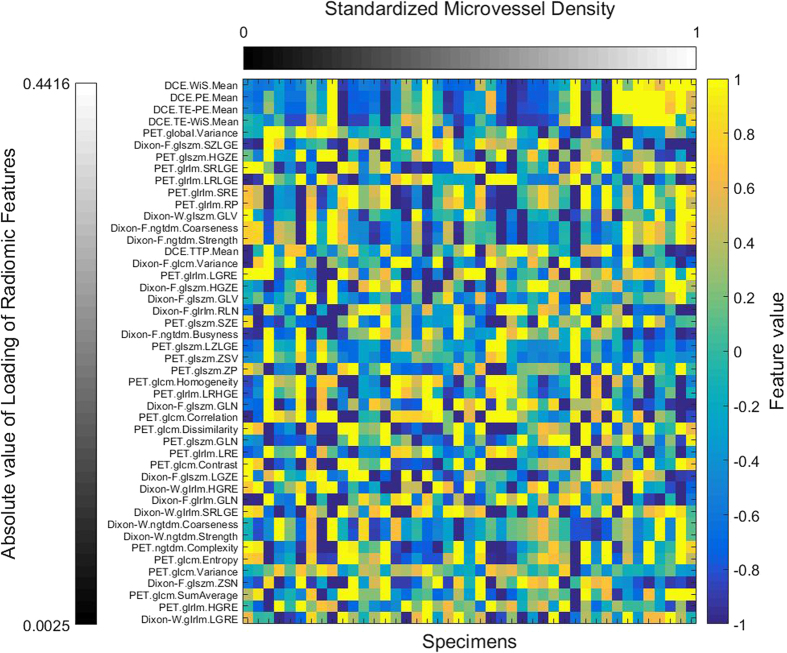
Heat map showing the association of the radiomic feature expression and angiogenesis, which are from the 1^st^ pair of canonical component. The columns represent specimens which are sorted from right to left according to the Microvessel density, and rows represent the radiomic features which loadings are not equal to zero in canonical component, and are from up to down in descending order according to the absolute value.

**Figure 8:**
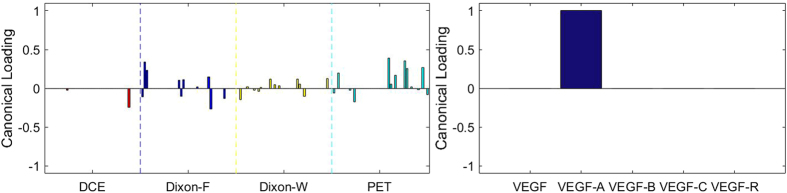
The 1^st^ canonical component for sparse canonical correlation analysis between radiomic features and VEGF (*p* = 0.169, z-stat) with spearman correlation coefficient *ρ*_1_ = 0.756.

**Figure 9:**
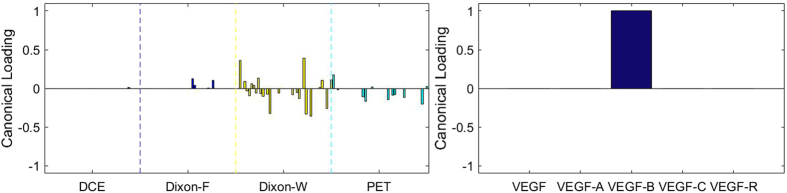
The 2^nd^ canonical component for sparse canonical correlation analysis between radiomic features and VEGF (*p* = 0.169, z-stat) with spearman correlation coefficient *ρ*_2_ = 0.603.

**Figure 10:**
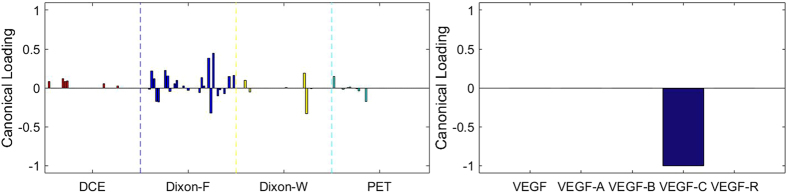
The 3^rd^ canonical component for sparse canonical correlation analysis between radiomic features and VEGF (*p* = 0.169, z-stat) with spearman correlation coefficient *ρ*_3_ = 0.674.

**Figure 11:**
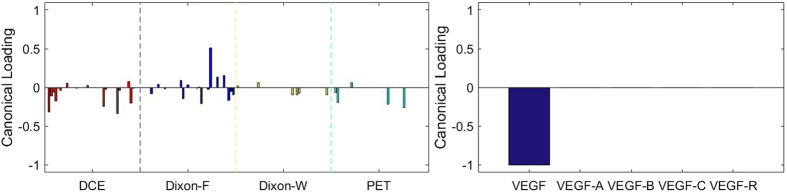
The 4^th^ canonical component for sparse canonical correlation analysis between radiomic features and VEGF (*p* = 0.169, z-stat) with spearman correlation coefficient *ρ*_4_ = 0.743.

**Figure 12:**
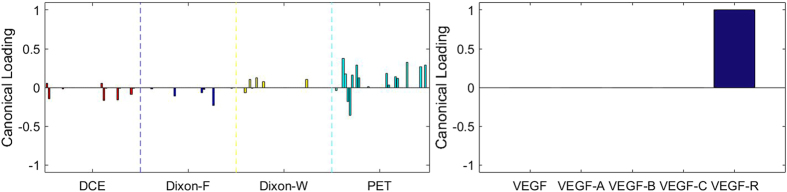
The 5^th^ canonical component for sparse canonical correlation analysis between radiomic features and VEGF (*p* = 0.169, z-stat) with spearman correlation coefficient *ρ*_5_ = 0.539.

Figures 3, 4, 5, 6, 7, 8, 9, 10, 11, 12 show the results of the canonical correlation analysis, which demonstrate the relationship (i.e., linear combination of weights) among three sets of variables, including radiomic features, MVD, and VEGF family. The height of each bar shows the correlation of each variable with the corresponding set of canonical weights. The canonical correlation coefficient (*ρ*) for each pair of linear composites is shown in the legends of each pair, representing the relationship between the two sets of variable (radiomic features *vs.* MVD, or radiomic features *vs.* VEGF family).

Based on the above descriptions, it is known that *h* = 4 pairs of canonical components can be extracted. As shown in Figs 3, 4, 5, 6, with significance level of *p* = 0.044, there are 4 clear and explicable relationships between MVD and RF with 43 specimens, which correlation coefficient are in Table 1 with the canonical components of total MVD, small-sized, medium-sized, and large-sized MVD, respectively.

In Figs 3, 4, 5, 6, it can be observed that the effects of DCE, Dixon_F and PET of RF dominate all four pairs of canonical components. The 1^st^, 13^rd^, 25^th^ and 31^st^ features (i.e., PE.Mean, WiS.Mean, TE_PE.Mean and TE_WiS.Mean in DCE) are the most consistent dominant weight vectors of RF in all canonical components. Other dominant features include the 74^th^ feature (i.e., GLSZM.SZLGE in Dixon_F) in total MVD, MVD of small-sized and medium-sized MVD, the 127^th^ feature (i.e., GLOBAL.Variance in PET), in small-sized, medium-sized MVD, and the 53^rd^ feature (i.e., GLCM.Variance in Dixon_F), the 102^nd^ feature (i.e., GLRLM.HGRE in Dixon_W), and the 151^st^ feature (i.e., GLSZM.SZE in PET) in large-sized MVD. The heap map in Fig. 7 shows the association of the radiomic feature expression and angiogenesis, which are from the 1^st^ pair of canonical components. The columns represent specimens, and the rows represent the radiomic features with their loadings not equal to zero in canonical component.

### Identifying PET/MRI Features Associated with Angiogenesis

In Table 1, we have summarized the results of SCCA using different group of features. Only groups of features extracted from DCE and PET/MRI achieved significant correlations between radiomic features and MVD. There were no significant correlations between MVD and the features from MRI or PET alone.

The correlation coefficients are the highest in the PET/MRI group, which incorporate both radiomic features from PET and MRI, than DCE alone, MRI alone and PET alone.

### Sparse Canonical Correlation Analysis between RF and *VEGF* set with 23 Specimens

There are at most *h* = 5 pairs of canonical components that can be extracted. As shown in Figs 8, 9, 10, 11, 12, there are 5 relationships between RF and *VEGF s*et. However, the relationships are not significant (*p* = 0.169 for z-stat).

Although the results were not significant, it is noticeable that the temporal features of DCE are not the most contributing features to all of the VEGF family. There are different trends of contributions of imaging features to each component of VEGF family. Total VEGF expressions were associated with features of DCE, Dixon_F and PET images. VEGF-A expressions, which is the main component of VEGF family and stimulates angiogenesis in health and diseases^11^, were more associated with Dixon_F and PET images. The effects of VEGF-B on pathological angiogenesis were less pronounced, and the deficiency of VEGF-B in mice does not impair angiogenesis in normal development^39^. In our study, VEGF-B expressions were more associated with RF of Dixon_W, which is different from the patterns of other members of VEGF family. One of the receptors of VEGF-C, VEGF-R3, is crucial for formation of the blood vasculature during early embryogenesis, and also important in later formation of new lymphatic vessels^40^. VEGF-C expressions were associated with both Dixon_F and Dixon_W, and expressions of VEGF receptor were most associated with Dixon_F and PET images.

## Discussion

One of the major advantages of this study was that the pathological tissue sections, from which MVD and expressions of VEGF family were measured, were all carefully extracted from the pre-defined ROIs, which provides an opportunity to perform region-to-region histological correlations of abstract radiomics features. Our results demonstrated several findings. First, we successfully identified the significant correlations between radiomic features and MVD. Second, spatiotemporal features extracted from DCE provide the highest loadings for explanation of the radiomic correlations of MVD than textural features from Dixon sequences and FDG PET. Third, taking advantage of the combined functional and structural information of FDG PET and MRI, this study has shown higher radiomics correlation of MVD than solely utilizing PET or MRI alone.

The four most consistently dominant features (loading >0.3) correlated with MVD were all spatiotemporal features of DCE about rapid gadolinium uptake, including absolute and relative peak enhancement and also wash-in slopes after gadolinium enhancement, which are similar with previous parameters described in breast, liver and colorectal cancers^41^,^42^,^43^,^44^,^45^,^46^,^47^. Although early tumor washout, probably associated with leaky capillaries, has also been proposed to be correlated with MVD, we were not able to find such significant associations between washout slope features and MVD.

The advantages of PET/MRI include shorter acquisition time of PET and MR images, which can increase the toleration of participants, and also simultaneous acquisition minimizes the chances of interscan motion and inevitable variation of tumor conditions. The ccRCC is characteristic of intracytoplasmic lipid accumulation. In this study, in addition to spatiotemporal features from DCE, we also extracted additional features from the Dixon sequence, which can separate the fat and water components of tumors and has been proposed to differentiate benign and malignant renal tumor using signal intensity drop in chemical-shift MRI^48^. As shown in Table 1, compared with using DCE images alone, additional features of Dixon contributed to a small improvement of correlation. As to glucose metabolism, ccRCCs utilize aerobic glycolysis in a similar way of ATP production like many other cancers, which are reflected by increased uptake on ^18^F-FDG PET^49^. Although our results showed the PET features had no significant correlations with MVD, compared with DCE-MRI features, a combined dual-modality features of PET/MRI achieved the highest performance of radiomic correlations with MVD.

VEGF is thought to play a major role in tumor angiogenesis. Although a decrease in permeability parameter, *K*_*trans*_, has been described in DCE-MRI parameters after administration of anti-VEGFR agents^50^, no studies, utilizing imaging for assessing the vascular features of renal tumors, had identified significant correlations between VEGF expressions and kinetic CT or MRI parameters^16^,^51^. Our second part of the radiomic analysis also did not identify significant associations between imaging features and VEGF family expressions. On the other hand, we observed a significant association with MVD, which represents an important functional metric of VEGF signaling in renal tumors. The potential value of the ability to assess tumors spatially with regard to functional features like MVD is 1) providing potential additional prognostic information, 2) adding information that may be valuable in predicting a diagnosis of disease that is *either* benign *or* indolent vs. malignant and with metastatic potential, and 3) guiding biopsy or surgical planning. This opportunity, in an era of rapid expansion in precision treatment selection, provides a potentially highly valuable piece of information, particularly in the selection of therapy that is likely to include anti-angiogenic agents. Finally, the evaluation of disease state for systemic therapy planning will allow physicians to assess disease holistically, rather than from just the end of a single needle. But, further studies with a large sample size are certainly necessary to investigate the trends we observed, and also the integration studies in which anti-angiogenic agents are administered will be an important future consideration.

There are several limitations in this study. First, in 47% of the samples (20/43), there were insufficient specimens for measurement of VEGF expressions, which may be insufficient to assess the associations of radiomic features. Further investigation on more cases may improve the statistical power. Second, the temporal resolution of multiphasic imaging is lower than the 5-second or 6-second temporal resolution in other DCE studies, which can more precisely determine the wash-in and wash-out parameters^18^,^52^. Higher temporal resolution will likely improve the utility of spatiotemporal features. Third, in order to improve the accuracy of image registration, we only utilized Dixon and DCE images, which provide 3D highresolution isotropic images, but we did not include other commonly-used 2D sequences, including T2-weighted MRI and diffusion-weighted MRI, which have thicker slices and are thus sensitive to patient motion between slices.

In summary, our analysis is a step forward towards highlighting the clinical advantage of hybrid PET/MRI and radiomics-based clinical interpretation. This study has utilized radiomics analysis to correlate with angiogenesis of primary ccRCCs using PET/MRI. Our results suggested that PET/MRI radiomic features, including peak enhancement and wash-in slope, might be able to quantify MVD in primary ccRCC. The results, obtained in this study, are just the basis to predict intratumor genomic heterogeneity in the future. In learning (classification or regression) models, the learning accuracy and training speed may be significantly deteriorated by those superfluous features. So, it is of fundamental importance to select the relevant and necessary features in the preprocessing step^53^. A good feature subset is one that contains features highly correlated with the class yet uncorrelated with each other^54^. In this sense, SCCA is a good method for feature selection because SCCA seek a sparse solution of CCA by incorporating variable selections to cope with the multi-collinearity within each dataset, and maximize the mutual information between the two datasets^55^,^56^. But our conclusions are limited by the small size of samples used. With expanding radiomics cohort and feature dimensions, we expect higher correlation in the future radiomics studies. Also, further investigation is needed to validate our findings on a larger cohort of data. Through our presented workflow, we also hope to extend the application of radiomics analysis to predicting intratumor genomic heterogeneity in the future.

## Additional Information

**How to cite this article**: Yin, Q. *et al*. Associations between Tumor Vascularity, Vascular Endothelial Growth Factor Expression and PET/MRI Radiomic Signatures in Primary Clear-Cell–Renal-Cell-Carcinoma: Proof-of-Concept Study. *Sci. Rep.* **7**, 43356; doi: 10.1038/srep43356 (2017).

**Publisher's note:** Springer Nature remains neutral with regard to jurisdictional claims in published maps and institutional affiliations.

## Supplementary Material

Supplementary Information

## Figures and Tables

**Table 1:** Correlation coefficients of microvascular density of different vessel sizes (in rows) and sets of radiomic features selected for sparse canonical correlation analysis (in column).

Microvessel density	PET/MRI		DCE alone		MRI alone (DCE + Dixon)		PET alone	
	Pearson correlation	Spearman Correlation	Pearson correlation	Spearman Correlation	Pearson correlation	Spearman Correlation	Pearson correlation	Spearman Correlation
Whole	0.639257121	0.466324373	0.538858721	0.340984597	0.531014484	0.347780127	0.43974722	0.440652371
small	0.634724358	0.375415282	0.542388517	0.365297493	0.532341511	0.366807611	0.42893314	0.367411658
middle	0.626200618	0.512836001	0.501232994	0.306704923	0.492377955	0.315010571	0.441627902	0.455451525
large	0.608610984	0.655693144	0.452889697	0.255964965	0.527560997	0.327393537	0.282646961	0.298701299
*p* value (Z-stat)	0.044		0.048		0.084		0.26	
