# Supplementary material for: Associations between Tumor Vascularity, Vascular Endothelial Growth Factor Expression and PET/MRI Radiomic Signatures in Primary Clear-Cell–Renal-Cell-Carcinoma: Proof-of-Concept Study
Source: Sci Rep. 2017 Mar 3;7:43356. doi: 10.1038/srep43356 (PMC5335708; doi:10.1038/srep43356)
Supplement: Supplementary Information [file srep43356-s1.pdf]

Associations between Tumor Vascularity, Vascular Endothelial Growth Factor Expression and PET/MRI Radiomic Signatures in Primary Clear-Cell–Renal-Cell-Carcinoma: Proof-of-Concept Study

Qingbo Yin, Ph.D.[1, 5, +], Sheng-Che Hung, M.D.[2, 3, 4, +], Li Wang, Ph.D.[5], Weili Lin, Ph.D.[5], Julia R. Fielding, M.D.[6], W. Kimryn Rathmell, M.D., Ph.D.[7,8,9,10], Amir H. Khandani, M.D.[5], Michael E. Woods, M.D., FACS[7,11], Matthew I. Milowsky, M.D.[7,8,11], Samira A. Brooks, Ph. D.[7], Eric. M. Wallen, M.D., FACS[11], Dinggang Shen, Ph.D.[5, 12,*]

[1] College of Information Science and Technology, Dalian Maritime University, Dalian, 116023, China

[2] Department of Radiology, Taipei Veterans General Hospital, Taipei, Taiwan 11217

[3] School of Medicine, National Yang-Ming University, Taipei, Taiwan 11221

[4] Department of Biomedical Imaging and Radiological Sciences, School of Biomedical Science of Engineering, National Yang-Ming University, Taipei, Taiwan 11221

[5] Department of Radiology, University of North Carolina, Chapel Hill, NC 27599

[6] Department of Radiology, University of Texas Southwestern Medical Center, Dallas, TX 75390

[7] Lineberger Comprehensive Cancer Center, University of North Carolina, Chapel Hill, NC 27599

[8] Department of Medicine, University of North Carolina, Chapel Hill, NC 27599

[9] Department of Genetics, University of North Carolina, Chapel Hill, NC 27599,

[10] Vanderbilt Ingram Cancer Center, Vanderbilt University, Nashville, TN 37232

[11] Department of Urology, University of North Carolina, Chapel Hill, NC 27599

[12] Department of Brain and Cognitive Engineering, Korea University, Seoul 02841, Republic of Korea

+ Co-first authors

*Correspondence and requests for materials should be addressed to Dinggang Shen (email: dgshen@med.unc.edu)

Supplement Tables

Table S1. Magnetic resonance imaging protocol for renal tumors

Pulse Sequences	Dixon for MR AC	3D Dixon	DCE 3D VIBE
**Plane**	coronal	axial	axial
**Duration (sec)**	19	19	16
**TE (IP/OP)*; TR (msec)**	(2.46/1.23); 3.6	(2.46/3.69); 5.48	1.88; 4.07
**Flip angle (degrees)**	10	9	9
**Bandwidth (Hz)**	965	504	446
**Number of excitation**	1	1	1
**Matrix size**	192 x 126	240 x 320	260 x 320
**Field of view (cm)**	50 x 32.8	28.5 x 38	32.5 x 40
**Slice thickness (mm)**	3.12	3	3.5

AC: attenuation correction; DCE: dynamic contrast enhancement; IP: in phase;

OP: opposed phase; TE: echo time; TR: repetition time;

VIBE: volumetric interpolated breath-hold examination

Table S2. The list of Radiomics Features

Index	Modality	Feature name
1	DCE	PE.Mean
2		PE.M21
3		PE.M22
4		PE.M23
5		PE.M3
6		PE.M4
7		TTP.Mean
8		TTP.M21
9		TTP.M22
10		TTP.M23
11		TTP.M3
12		TTP.M4
13		WiS.Mean
14		WiS.M21
15		WiS.M22
16		WiS.M23
17		WiS.M3
18		WiS.M4
19		WoS.Mean
20		WoS.M21
21		WoS.M22
22		WoS.M23
23		WoS.M3
24		WoS.M4
25		TE_PE.Mean
26		TE_PE.M21
27		TE_PE.M22

28		TE_PE.M23
29		TE_PE.M3
30		TE_PE.M4
31		TE_WiS.Mean
32		TE_WiS.M21
33		TE_WiS.M22
34		TE_WiS.M23
35		TE_WiS.M3
36		TE_WiS.M4
37		TE_WoS.Mean
38		TE_WoS.M21
39		TE_WoS.M22
40		TE_WoS.M23
41		TE_WoS.M3
42		TE_WoS.M4
43	Dixon-F	global.variance
44		global.skewness
45		global.Kurtosis
46		glcm.Energy
47		glcm.Contrast
48		glcm.Entropy
49		glcm.Homogeneity
50		glcm.Correlation
51		glcm.SumAverage
52		glcm.Variance
53		glcm.Dissimilarity
54		glrlm.SRE
55		glrlm.LRE
56		glrlm.GLN
57		glrlm.RLN
58		glrlm.RP
59		glrlm.LGRE
60		glrlm.HGRE
61		glrlm.SRLGE
62		glrlm.SRHGE
63		glrlm.LRLGE
64		glrlm.LRHGE
65		glrlm.GLV
66		glrlm.RLV
67		glszm.SZE

68		glszm.LZE
69		glszm.GLN
70		glszm.ZSN
71		glszm.ZP
72		glszm.LGZE
73		glszm.HGZE
74		glszm.SZLGE
75		glszm.SZHGE
76		glszm.LZLGE
77		glszm.LZHGE
78		glszm.GLV
79		glszm.ZSV
80		ngtdm.Coarseness
81		ngtdm.Contrast
82		ngtdm.Busyness
83		ngtdm.Complexity
84		ngtdm.Strength
85	Dixon-W	global.Variance
86		global.Skewness
87		global.Kurtosis
88		glcm.Energy
89		glcm.Contrast
90		glcm.Entropy
91		glcm.Homogeneity
92		glcm.Correlation
93		glcm.SumAverage
94		glcm.Variance
95		glcm.Dissimilarity
96		glrlm.SRE
97		glrlm.LRE
98		glrlm.GLN
99		glrlm.RLN
100		glrlm.RP
101		glrlm.LGRE
102		glrlm.HGRE
103		glrlm.SRLGE
104		glrlm.SRHGE
105		glrlm.LRLGE
106		glrlm.LRHGE
107		glrlm.GLV

108		glrlm.RLV
109		glszm.SZE
110		glszm.LZE
111		glszm.GLN
112		glszm.ZSN
113		glszm.ZP
114		glszm.LGZE
115		glszm.HGZE
116		glszm.SZLGE
117		glszm.SZHGE
118		glszm.LZLGE
119		glszm.LZHGE
120		glszm.GLV
121		glszm.ZSV
122		ngtdm.Coarseness
123		ngtdm.Contrast
124		ngtdm.Busyness
125		ngtdm.Complexity
126		ngtdm.Strength
127	PET	global.Variance
128		global.Skewness
129		global.Kurtosis
130		glcm.Energy
131		glcm.Contrast
132		glcm.Entropy
133		glcm.Homogeneity
134		glcm.Correlation
135		glcm.SumAverage
136		glcm.Variance
137		glcm.Dissimilarity
138		glrlm.SRE
139		glrlm.LRE
140		glrlm.GLN
141		glrlm.RLN
142		glrlm.RP
143		glrlm.LGRE
144		glrlm.HGRE
145		glrlm.SRLGE
146		glrlm.SRHGE
147		glrlm.LRLGE

148		glrlm.LRHGE
149		glrlm.GLV
150		glrlm.RLV
151		glszm.SZE
152		glszm.LZE
153		glszm.GLN
154		glszm.ZSN
155		glszm.ZP
156		glszm.LGZE
157		glszm.HGZE
158		glszm.SZLGE
159		glszm.SZHGE
160		glszm.LZLGE
161		glszm.LZHGE
162		glszm.GLV
163		glszm.ZSV
164		ngtdm.Coarseness
165		ngtdm.Contrast
166		ngtdm.Busyness
167		ngtdm.Complexity
168		ngtdm.Strength

Supplement Method: The Definition of Radiomics Features

These radiomic features described the tumor characteristics and can be divided into two groups: group 1, spatiotemporal association features (ST_F) and group 2, textures features (TEX_F). Group 1 included 42 features extracted from the time-series modality DCE-MRI, which represent the tumor spatiotemporal enhancement pattern within each ROI. Group 2 consisted of 42 x 3 textural features that quantified the intra-tumor heterogeneity differences in the textures of tumor volume contained within each ROI. Group 2 were obtained from the non-time-series modalities (Dixon_F, Dixon_W and PET), in which modality 42 features are obtained.

Group 1. Spatiotemporal association features (ST_F)

A. Subgroup: kinetic features in DCE-MRI

Let $V(x, y, z)$ represent a voxel in the ROI.

Various kinetic features were calculated for each voxel within a tumor, involve temporal enhancement (TE) at each temporal phase and peak enhancement (PE) and time-to-peak (TTP) and wash-in slope (WiS) and wash-out slope (WoS).

The temporal enhancement can be obtained by

$$TE_t = \frac{V_t - V_0}{V_0},\ 0 < t \leq M$$

Where t denotes the time elapsed from contrast injection and M is the elapsed time of the last temporal phase. V_0 and V_t indicate the precontrast and postcontrast signal intensity taken at time t, respectively.

Peak enhancement (PE)

$$PE = \max_{0<t\leq M} TE_t$$

Time-to-peak (TTP)

$$TTP = \arg\max_{0<t\leq M} TE_t$$

Wash-in slope (WiS)

$$\mathrm{W_I S} = \frac{PE}{TTP}$$

Wash-out slope (WoS)

$$WoS = \begin{cases} \dfrac{PE - TE_M}{M - TTP} & (TTP \neq M) \\ 0 & (TTP = M) \end{cases}$$

TE_PE denotes to calculate TE on the result matrix of PE.

TE_WiS denotes to calculate TE on the result matrix of WiS.

TE_WoS denotes to calculate TE on the result matrix of WoS.

B. Subgroup: spatial features

As for a 3D object, we can define

$$m_{pqr} = \iiint x^p y^q z^r \rho(x, y, z) dxdydz$$

$$\mu_{pqr} = \iiint (x - \bar{x})^p (y - \bar{y})^q (z - \bar{z})^r \rho(x, y, z) dxdydz$$

$$\bar{x} = \frac{m_{100}}{m_{000}}\ , \quad \bar{y} = \frac{m_{010}}{m_{000}}, \quad \bar{z} = \frac{m_{001}}{m_{000}},$$

$$\eta_{pqr} = \frac{\mu_{pqr}}{\mu_{000}^{[(p+q+r)/3]+1}}$$

1) M_{21}

$$M_{21} = \eta_{200} + \eta_{020} + \eta_{002}$$

2) M_{22}

$$M_{22} = \eta_{200}\eta_{020} + \eta_{200}\eta_{002} + \eta_{020}\eta_{002} - \eta_{101}^2 - \eta_{110}^2 - \eta_{011}^2$$

3) M_{23}

$$M_{23} = \eta_{200}\eta_{020}\eta_{002} - \eta_{002}\eta_{110}^2 + 2\eta_{110}\eta_{101}\eta_{011} - \eta_{020}\eta_{101}^2 - \eta_{200}\eta_{011}^2$$

4) M_3

$$M_3 = \eta_{300}^2 + \eta_{030}^2 + \eta_{003}^2 + 3\eta_{210}^2 + 3\eta_{201}^2 + 3\eta_{120}^2 + 6\eta_{111}^2 + 3\eta_{102}^2 + 3\eta_{021}^2 + 3\eta_{012}^2$$

5) M_4

$$M_4 = \eta_{400}^2 + \eta_{040}^2 + \eta_{004}^2 + 4\eta_{310}^2 + 4\eta_{301}^2 + 6\eta_{220}^2 + 12\eta_{211}^2 + 6\eta_{202}^2 + 4\eta_{130}^2 + 12\eta_{121}^2 + 12\eta_{112}^2 + 4\eta_{103}^2 + 4\eta_{031}^2 + 6\eta_{022}^2 + 4\eta_{013}^2$$

C. Definition of variables(features) in ROI

PE.Mean: mean of PE

TTP.Mean: mean of TTP

WiS.Mean: mean of WiS

WoS.Mean: mean of WoS

TE_PE.Mean: mean of TE_PE

TE_TTP.Mean: mean of TE_TTP

TE_WiS.Mean: mean of TE_WiS

TE_WoS.Mean: mean of TE_WoS

Other features in this part follow the same rules.

Group 2. Textures features (TEX_F)

A. Subgroup: Global

1) Variance variance $= \frac{1}{N-1}\sum_{i=1}^{N}(x(i) - \overline{x})^2$,

$\overline{x}$ is the mean of x.

2) Skewness

$$\text{Skewness} = \frac{\frac{1}{N}\sum_{i=1}^{N}(x(i)-\overline{x})^3}{\left(\sqrt{\frac{1}{N}\sum_{i=1}^{N}(x(i)-\overline{x})^2}\right)^3}$$

3) Kurtosis

$$\text{kurtosis} = \frac{\frac{1}{N}\sum_{i=1}^{N}(x(i)-\overline{x})^4}{\left(\sqrt{\frac{1}{N}\sum_{i=1}^{N}(x(i)-\overline{x})^2}\right)^2}$$

B. Subgroup: Gray level Co-occurrence Matrix

Let P define the GLCM of a quantized volume $V(x,y,z)$ with isotropic voxel size.

$P(i,j)$: the number of times which voxels of gray-level i were neighbors with voxels of the gray level j in V.

N_g: the number of discrete intensity value in V.

$p(i,j)$: the $(i,j)^{\text{th}}$ entry in the normalized GLCM P.

$$p(i,j) = \frac{P(i,j)}{\sum_{i=1}^{N_g}\sum_{j=1}^{N_r}P(i,j)}$$

N_p: the number of voxels in the V.

$\mu_i = \sum_{i=1}^{N_g} i \sum_{j=1}^{N_g} p(i,j)$ $\quad\mu_j = \sum_{j=1}^{N_g} j \sum_{i=1}^{N_g} p(i,j)$

$\sigma_i = \sum_{i=1}^{N_g}(i-\mu_i)\sum_{j=1}^{N_g} p(i,j)$ $\quad\sigma_j = \sum_{j=1}^{N_g}(j-\mu_j)\sum_{i=1}^{N_g} p(i,j)$

4) Energy

$$\text{energy} = \sum_{i=1}^{N_g}\sum_{i=1}^{N_g}[p(i,j)]^2$$

5) Contrast

$$\text{contrast} = \sum_{i=1}^{N_g} \sum_{i=1}^{N_g} [i-j]^2 p(i,j)$$

6) Correlation

$$\text{correlation} = \frac{\sum_{i=1}^{N_g} \sum_{i=1}^{N_g} (i-\mu_i)(j-\mu_j) p(i,j)}{\sigma_i \sigma_j}$$

7) Homogeneity

$$\text{homogeneity} = \sum_{i=1}^{N_g} \sum_{i=1}^{N_g} \frac{p(i,j)}{1+|i-j|^2}$$

8) Variance

$$\text{variance} = \frac{1}{N_g \times N_g} \sum_{i=1}^{N_g} \sum_{j=1}^{N_g} \left[(i-\mu_i)^2 + \left(i-\mu_j\right)^2\right] p(i,j)$$

9) Sum Average

$$\text{sum average} = \frac{1}{N_g \times N_g} \sum_{i=1}^{N_g} \sum_{j=1}^{N_g} [i+j] p(i,j)$$

10) Entropy

$$\text{entropy} = -\sum_{i=1}^{N_g} \sum_{i=1}^{N_g} p(i,j) log_2 p(i,j)$$

11) Dissimilarity

$$\text{dissimilarity} = \sum_{i=1}^{N_g} \sum_{i=1}^{N_g} |i-j| p(i,j)$$

C. Subgroup: Gray-level run-length matrix

Use of gray value distribution of run lengths for texture analysis

The analysis of natural textures using run length features

Texture analysis using gray level run lengths

Let P define the GLRLM of a quantized volume $V(x, y, z)$ with isotropic voxel size.

$P(i, j|\theta)$: the number of runs of gray-level i and of length j for a direction θ in V.

$p(i, j|\theta)$: the $(i, j)^{\text{th}}$ entry in the normalized GLRLM P for a direction θ.

$$p(i, j|\theta) = \frac{P(i, j|\theta)}{\sum_{i=1}^{N_g} \sum_{j=1}^{N_r} P(i, j|\theta)}$$

N_g: the number of discrete intensity value in V.

N_r: the number of different run lengths

N_p: the number of voxels in the V.

$\mu_i = \sum_{i=1}^{N_g} i \sum_{j=1}^{N_r} p(i, j|\theta)$ $\mu_j = \sum_{j=1}^{N_r} j \sum_{i=1}^{N_g} p(i, j|\theta)$

12) Short run emphasis (SRE)

$$\text{SRE} = \sum_{i=1}^{N_g} \sum_{j=1}^{N_r} \left[\frac{p(i, j|\theta)}{j^2} \right]$$

13) Long run emphasis (LRE)

$$\text{LRE} = \sum_{i=1}^{N_g} \sum_{j=1}^{N_r} j^2 p(i, j|\theta)$$

14) Gray level non-uniformity(GLN)

$$\text{GLN} = \sum_{i=1}^{N_g} \left[\sum_{j=1}^{N_r} p(i, j|\theta) \right]^2$$

15) Run length non-uniformity(RLN)

$$\mathrm{RLN} = \sum_{j=1}^{N_r}\left[\sum_{i=1}^{N_g} p(i,j|\theta)\right]^2$$

16) Run Percentage(RP)

$$\mathrm{RP} = \frac{\sum_{i=1}^{N_g}\sum_{j=1}^{N_r} p(i,j|\theta)}{\sum_{j=1}^{N_r} j \sum_{i=1}^{N_g} p(i,j|\theta)}$$

17) Low gray-level run emphasis(LGLRE)

$$\mathrm{LGLRE} = \sum_{i=1}^{N_g}\sum_{j=1}^{N_r}\left[\frac{p(i,j|\theta)}{i^2}\right]$$

18) High gray-level run emphasis(HGLRE)

$$\mathrm{HGLRE} = \sum_{i=1}^{N_g}\sum_{j=1}^{N_r} i^2 p(i,j|\theta)$$

19) Short run low gray-level emphasis(SRLGLE)

$$\mathrm{SRLGLE} = \sum_{i=1}^{N_g}\sum_{j=1}^{N_r}\left[\frac{p(i,j|\theta)}{i^2 j^2}\right]$$

20) Short run high gray-level emphasis(SRHGLE)

$$\mathrm{SRHGLE} = \sum_{i=1}^{N_g}\sum_{j=1}^{N_r}\left[\frac{i^2 p(i,j|\theta)}{j^2}\right]$$

21) Long run low gray-level emphasis(LRLGLE)

$$\mathrm{LRLGLE} = \sum_{i=1}^{N_g}\sum_{j=1}^{N_r}\left[\frac{p(i,j|\theta) j^2}{i^2}\right]$$

22) Long run high gray-level emphasis(LRHGLE)

$$\mathrm{LRHGLE} = \sum_{i=1}^{N_g}\sum_{j=1}^{N_r} p(i,j|\theta) i^2 j^2$$

23) Gray level variance(GLV)

$$\mathrm{GLV} = \frac{1}{N_g \times N_r} \sum_{i=1}^{N_g} \sum_{j=1}^{N_r} (ip(i,j|\theta) - \mu_i)^2$$

24) Run length Variance(RLV)

$$\mathrm{RLV} = \frac{1}{N_g \times N_r} \sum_{i=1}^{N_g} \sum_{j=1}^{N_r} \left(jp(i,j|\theta) - \mu_j\right)^2$$

D. Subgroup: Gray level size zone matrix

Let P define the GLSZM of a quantized volume $V(x,y,z)$ with isotropic voxel size.

$P(i,j)$: the number of 3D zones of gray-level i and of size j in V.

$p(i,j)$: the $(i,j)^{\mathrm{th}}$ entry in the normalized GLSZM P.

$$p(i,j) = \frac{P(i,j)}{\sum_{i=1}^{N_g} \sum_{j=1}^{N_r} P(i,j)}$$

N_g: the number of quantized gray-level set in V.

N_z: the size of the largest zone in V.

N_p: the number of voxels in the V.

$\mu_i = \sum_{i=1}^{N_g} i \sum_{j=1}^{N_z} p(i,j)$ $\mu_j = \sum_{j=1}^{N_z} j \sum_{i=1}^{N_g} p(i,j)$

25) Small zone emphasis (SZE)

$$\mathrm{SZE} = \sum_{i=1}^{N_g} \sum_{j=1}^{N_z} \left[\frac{p(i,j)}{j^2}\right]$$

26) Large zone emphasis (LZE)

$$\mathrm{LZE} = \sum_{i=1}^{N_g} \sum_{j=1}^{N_z} j^2 p(i,j)$$

27) Gray level non-uniformity (GLN)

$$\mathrm{GLN} = \sum_{i=1}^{N_g} \left[\sum_{j=1}^{N_z} p(i,j) \right]^2$$

28) Zone size non-uniformity (ZSN)

$$\mathrm{ZSN} = \sum_{j=1}^{N_z} \left[\sum_{i=1}^{N_g} p(i,j) \right]^2$$

29) Zone percentage (ZP)

$$\mathrm{ZP} = \frac{\sum_{i=1}^{N_g} \sum_{j=1}^{N_z} p(i,j)}{\sum_{j=1}^{N_z} j \sum_{i=1}^{N_g} p(i,j)}$$

30) Low gray level zone emphasis (LGZE)

$$\mathrm{LGZE} = \sum_{i=1}^{N_g} \sum_{j=1}^{N_z} \left[\frac{p(i,j)}{i^2} \right]$$

31) High gray level zone emphasis (HGZE)

$$\mathrm{HGZE} = \sum_{i=1}^{N_g} \sum_{j=1}^{N_r} i^2 p(i,j)$$

32) Small zone low gray level emphasis (SZLGE)

$$\mathrm{SZLGE} = \sum_{i=1}^{N_g} \sum_{j=1}^{N_z} \left[\frac{p(i,j)}{i^2 j^2} \right]$$

33) Small zone high gray level emphasis (SZHGE)

$$\mathrm{SZHGE} = \sum_{i=1}^{N_g} \sum_{j=1}^{N_z} \left[\frac{i^2 p(i,j)}{j^2} \right]$$

34) Large zone low gray level emphasis (LZLGE)

$$\text{LZLGE} = \sum_{i=1}^{N_g}\sum_{j=1}^{N_r}\left[\frac{j^2 p(i,j|\theta)}{i^2}\right]$$

35) Large zone high gray level emphasis (LZHGE)

$$\text{LZHGE} = \sum_{i=1}^{N_g}\sum_{j=1}^{N_z} p(i,j|\theta) i^2 j^2$$

36) Gray level variance (GLV)

$$\text{GLV} = \frac{1}{N_g \times N_z}\sum_{i=1}^{N_g}\sum_{j=1}^{N_z}(ip(i,j) - \mu_i)^2$$

37) Zone size variance (ZSV)

$$\text{ZSV} = \frac{1}{N_g \times N_z}\sum_{i=1}^{N_g}\sum_{j=1}^{N_z}\left(jp(i,j|\theta) - \mu_j\right)^2$$

E. Neighborhood gray tone difference matrix (NGTDM)

Let P define the NGTDM of a quantized volume $V(x, y, z)$ with isotropic voxel size.

$P(i)$: the summation of the gray-level differences between all voxels with gray level i and the average gray level of their 26 connected neighbors in V.

N_g: the pre-defined number of quantized gray-level set in V.

$(N_g)_{eff}$: the effective number of gray levels in V.

One NGTDM of size $N_g \times 1$ is computed per volume V. To account for discretization length differences, all averages around a center voxel located at position (x,y,z) in V are performed such that the neighbors at a distance of $\sqrt{3}$ voxels are given a weight of $1/\sqrt{3}$, the neighbors at a distance of $\sqrt{2}$ voxels are given a weight of $1/\sqrt{2}$, and the neighbors at a distance of 1 voxel are given a weight of 1. The ith entry of the NGTDM is then defined as:

$$P(i) = \begin{cases} \sum_{all\ voxels \in \{N_i\}} |i - \bar{A}_i| & if\ N_i \neq 0 \\ 0 & otherwise \end{cases}$$

$\{N_i\}$ is the set of all voxels with gray level i in V. N_i is the number of voxels with gray level i in V, and $\bar{A}_i$ is the average gray level of the 26-connected neighbors around a center voxel with gray level i and located at position (x,y,z) in V such that

$$\bar{A}_i = \bar{A}(x,y,z) = \frac{\sum_{m=-1}^{1}\sum_{n=-1}^{1}\sum_{o=-1}^{1} V(x+m, y+n, z+o)}{\sum_{m=-1}^{1}\sum_{n=-1}^{1}\sum_{o=-1}^{1} w_{m,n,o}}$$

$$w_{m,n,o} = \begin{cases} 1 & if\ |x-m|+|y-n|+|z-o| = 1 \\ \frac{1}{\sqrt{2}} & if\ |x-m|+|y-n|+|z-o| = 2 \\ \frac{1}{\sqrt{3}} & if\ |x-m|+|y-n|+|z-o| = 3 \\ 0 & if\ V(x+m, y+n, z+o)\ is\ undefined \end{cases}$$

The following quantity is also defined

$$n_i = \frac{i}{N}$$

Where N is the total number of voxels in V. The NGTDM texture features are then defined as:

38) Coarseness

$$coarseness = \left[\epsilon + \sum_{i=1}^{N_g} n_i P(i)\right]^{-1}$$

Where ϵ is a small number to prevent *coarseness* becoming infinite.

39) Contrast

$$contrast = \left[\frac{1}{(N_g)_{eff}\left[(N_g)_{eff}-1\right]}\sum_{i=1}^{N_g}\sum_{j=1}^{N_g} n_i n_j (i-j)^2\right]\left[\frac{1}{N}\sum_{i=1}^{N_g} P(i)\right]$$

40) Busyness

$$busyness = \left[\frac{\sum_{i=1}^{N_g} n_i P(i)}{\sum_{i=1}^{N_g}\sum_{j=1}^{N_g}\left(i n_i - j n_j\right)}\right], \qquad n_i \neq 0, n_j \neq 0$$

41) Complexity

$$complexity = \sum_{i=1}^{N_g}\sum_{j=1}^{N_g}\frac{|i-j|\left[n_i P(i) + n_j P(j)\right]}{N\left(n_i + n_j\right)}, \qquad n_i \neq 0, n_j \neq 0$$

42) Strength

$$complexity = \frac{\sum_{i=1}^{N_g}\sum_{j=1}^{N_g}\left(n_i + n_j\right)(i-j)^2}{\left[\epsilon + \sum_{i=1}^{N_g} P(i)\right]}, \qquad n_i \neq 0, n_j \neq 0$$

Where ϵ is a small number to prevent *complexity* becoming infinite.
